# Multidrug-Resistant High-Risk *Escherichia coli* and *Klebsiella pneumoniae* Clonal Lineages Occur in Black-Headed Gulls from Two Conservation Islands in Germany

**DOI:** 10.3390/antibiotics11101357

**Published:** 2022-10-05

**Authors:** Jana Brendecke, Timo Homeier-Bachmann, Angela Schmitz Ornés, Sebastian Guenther, Stefan E. Heiden, Michael Schwabe, Elias Eger, Katharina Schaufler

**Affiliations:** 1 Pharmaceutical Microbiology, Institute of Pharmacy, University of Greifswald, 17489 Greifswald, Germany; 2 Institute of Epidemiology, Friedrich Loeffler-Institute, Federal Research Institute for Animal Health, 17493 Greifswald, Germany; 3 AG Vogelwarte, Zoological Institute and Museum, University of Greifswald, 17489 Greifswald, Germany; 4 Pharmaceutical Biology, Institute of Pharmacy, University of Greifswald, 17489 Greifswald, Germany; 5 Institute of Infection Medicine, Christian-Albrecht University Kiel and University Medical Center Schleswig-Holstein, 24105 Kiel, Germany

**Keywords:** antimicrobial resistance, black-headed gulls, next-generation sequencing, One Health, virulence, wildlife

## Abstract

Multidrug-resistant (MDR) *Enterobacterales*, including extended-spectrum β-lactamase (ESBL)-producing *Escherichia coli* and *Klebsiella pneumoniae*, not only emerge in healthcare settings but also in other habitats, such as livestock and wildlife. The spread of these pathogens, which often combine resistance with high-level virulence, is a growing problem, as infections have become increasingly difficult to treat. Here, we investigated the occurrence of ESBL-producing *E. coli* and *K. pneumoniae* in fecal samples from two black-headed gull colonies breeding on two nature conservation islands in Western Pomerania, Germany. In addition to cloacal samples from adult birds (*n* = 211) and their nestlings (*n* = 99) during the 2021 breeding season, collective fecal samples (*n* = 29) were obtained. All samples were screened for ESBL producers, which were then subjected to whole-genome sequencing. We found a total of 12 ESBL-producing *E. coli* and *K. pneumoniae* consisting of 11 *E. coli* and 1 *K. pneumoniae*, and including the international high-risk *E. coli* sequence types (ST)131, ST38, and ST58. Eight of the investigated strains had a MDR genotype and carried a large repertoire of virulence-associated genes, including the *pap* operon, which is important for urinary tract infections. In addition, we identified many genes associated with adherence, biofilm formation, iron uptake, and toxin production. Finally, our analysis revealed the close phylogenetic relationship of ST38 strains with genomes originating from human sources, underlining their zoonotic and pathogenic character. This study highlights the importance of the One Health approach, and thus the interdependence between human and animal health and their surrounding environment.

## 1. Introduction

Antibiotics such as the β-lactams revolutionized medicine, as infections that had previously been fatal became easily treatable. Nevertheless, nearly 100 years after the discovery of penicillin, reports of severe infections caused by multidrug-resistant (MDR) bacteria have accumulated [1]. For example, the high prevalence of extended-spectrum β-lactamase (ESBL)-producing *Enterobacterales* (e.g., *Escherichia coli* and *Klebsiella pneumoniae*) is a major public health issue [2,3] and also a veterinary and environmental concern [4,5]. Livestock, in particular, represent an important reservoir for MDR pathogens, which can then be transmitted to humans and other animals, including wildlife, via direct contact, sewage, manure, and the food chain [6,7,8]. It is therefore not surprising that we frequently find MDR *E. coli* and *K. pneumoniae* in various wildlife populations [9], including birds [10]. These Gram-negative bacteria have already been described in black-headed gull (*Chroicocephalus ridibundus*) colonies from Kalmar (Sweden) [11], Novi Sad (Serbia) [12], and different locations in the Czech Republic [13].

Previous studies suggested that a limited number of clonal lineages, such as *E. coli* sequence types (ST)131, ST38, ST617, ST648, ST744, and ST58 [14,15,16,17,18,19,20,21,22] and *K. pneumoniae* ST11, ST258, ST290, and ST307 [23,24] drive the global distribution of MDR pathogens. Due to the successful combination of virulence and antibiotic resistance, and the apparent lack of host specificity [24,25,26], they have the potential to cause infections in humans and animals. This also highlights the importance of the One Health approach, which emphasizes the inextricable linkage of human, animal (wild and domestic), and environmental health.

Here, we investigated the occurrence of ESBL-producing *E. coli* and *K. pneumoniae* in two remote black-headed gull colonies in Western Pomerania, Germany. Through whole-genome sequencing (WGS) and in-depth bioinformatics, this study aimed not only to determine (i) the prevalence of ESBL-producing *E. coli* and *K. pneumoniae* in the two gull colonies but also to characterize their (ii) phylogenetic background, as well as (iii) the resistance and virulence genes they carried.

## 2. Results

### 2.1. The Presence of ESBL-Producing E. coli and K. pneumoniae in Two Black-Headed Gull Colonies

**Figure 1:**
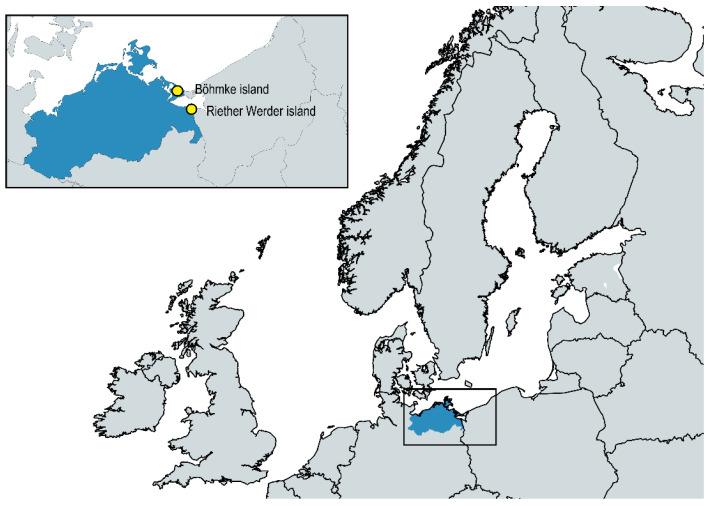
Both black-headed gull colonies are located on the German Baltic Sea coast. The large map shows the coastline of the Baltic Sea and the North Sea. Blue areas indicate the German federal state of Mecklenburg–Western Pomerania, where both black-headed gull colonies are located (yellow). The map was created using https://www.mapchart.net (accessed on 19 August 2022).

Sampling in both gull colonies took place during the breeding season (May–June 2021) on the islands of Böhmke and Riether Werder in Western Pomerania [27] (Figure 1).

We isolated 12 ESBL-producing strains from a total of 339 samples (3.5% (95% confidence interval (CI): 2.0–6.1%; 12 of 339)), consisting of 11 *E. coli* (3.2% (95% CI: 1.8–5.7%)) and 1 *K. pneumoniae* (0.3% (95% CI: 0–1.7%)). Interestingly, their distribution varied between the two sampling locations. We found most of the strains in samples from Böhmke Island (individual adult swabs, 2/100, 2.0%; individual nestling swabs, 4/99, 4.0%; collective fecal samples, 5/14, 35.7%); in contrast, only 1 of the 12 strains originated from a collective fecal sample from Riether Werder Island (1/15, 6.7%).

WGS revealed eight different STs (Table 1), with the presence of several representatives of the international high-risk lineages ST38 (*n* = 2), ST58 (*n* = 2), ST744 (*n* = 1), ST617 (*n* = 1) and ST131 (*n* = 1). Most strains (72.7%, 8/11) belonged to the *E. coli* phylogroups A and B1, mostly consisting of commensals and obligate pathogenic *E. coli* [28]. Other strains were assigned to phylogroups B2 (9.1%, 1/11) and D (18.2%, 2/11), which are associated with facultative extraintestinal pathogenic *E. coli* (ExPEC) [29]. The *K. pneumoniae* strain PBIO3691 belonged to ST290. The best matching capsule and O antigen loci of PBIO3691 were KL21 and O1/O2v1, with identities of ≥98.57% and ≥98.09%, respectively.

### 2.2. Resistance Profiles of the Isolated Strains

WGS analysis using the AMRFinderPlus database [30] revealed that *bla*_CTX-M-15_ (*n* = 7) and *bla*_CTX-M-14_ (*n* = 2) were the predominant ESBL genes and that five different CTX-M family members were present in total (Table 1). Note that the two ST38 strain as well as the ST58 strain carried different β-lactamase alleles, potentially indicating that they were unrelated. We then explored the context of the CTX-M-encoding genes further, as they are mostly carried by plasmids [31], but chromosomal integration has also been previously reported [32]. Interestingly, it appears that 4 of the 12 strains (PBIO3538, PBIO3541, PBIO3584, and PBIO3707) carried the ESBL gene chromosomally, with three of these strains belonging to the international high-risk clonal lineages ST38 (*n* = 2) and ST58 (*n* = 1), which is consistent with previous findings [33].

**Figure 2:**
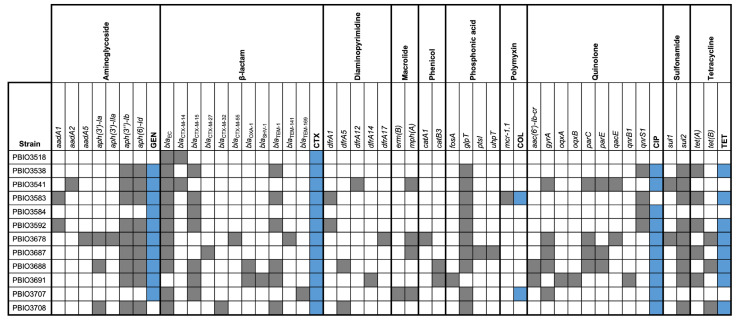
Geno- and phenotypic resistance of the 12 ESBL-producing strains. The corresponding gene content and resistant phenotypes are summarized in a presence–absence matrix. The predictions of resistance (genes and point mutations) (gray) were based on alignments of the sequences from the AMRFinderPlus database (default settings of identity ≥90.0% and coverage ≥50.0%). Resistant phenotypes (blue) were defined according to the published breakpoints for *Enterobacterales* of the Clinical and Laboratory Standards Institute (CLSI). Uncolored boxes indicate the absence of the genotype or phenotype. CIP, ciprofloxacin; COL, colistin; CTX, cefotaxime; GEN, gentamicin; TET, tetracycline.

In addition, all strains carried a BlaEC family class C β-lactamase (*bla*_EC_) (Figure 2). Note that genes encoding for carbapenemases (e.g., *bla*_OXA-48_ or *bla*_NDM-1_) were not present.

A growing number of studies have suggested that ESBL-producing *E. coli* and *K. pneumoniae* often exhibit co-resistance, resulting in multidrug-resistant (MDR) phenotypes [34,35]. In our study, 11 strains (91.7%, 11/12) carried genes or point mutations resulting in genotypic resistance to phosphonic acids (fosfomycin; *fosA*, *glpT*, *ptsI*, *uhpT*), followed by genes encoding resistance (resistance genes and point mutations) to quinolones (*aac(6′)-Ib-cr, gyrA*, *oqx*, *par*, *qacE*, and *qnr*; 83.3%, 10/12), aminoglycosides (*aad*, *ant*, and *aph*; 75.0%, 9/12), sulfonamides (*sul1*, *sul2*; 75.0%, 9/12), diaminopyrimidines (trimethoprim; *dfrA*; 58.3%, 7/12), macrolides (*erm* and *mph*; 33.3%, 4/12), and phenicols (*cat*; 25.0%, 3/12). One ST38 *E. coli* strain (PBIO3583) carried *mcr-1.1*, which confers resistance to colistin. In total, eight *E. coli* and one *K. pneumoniae* strain carried resistance genes to four or more different classes of antimicrobials and thus exhibited a MDR genotype [36].

To determine whether the genotype matched the phenotype, we then performed phenotypic antimicrobial susceptibility testing (AST) against ciprofloxacin (quinolones), gentamicin (aminoglycosides), tetracycline (tetracyclines), and colistin (polymyxins). In summary, the results of the phenotypic AST were mostly consistent with the genotype (Figure 2). Although PBIO3707 did not carry a typical *mcr* gene, it was phenotypically resistant to colistin (MIC, 4 μg/mL), similar to PBIO3583. We thus analyzed the genes related to lipopolysaccharide production, because it is known that modification of the respective enzymes can lead to colistin resistance [24]. Multiple sequence alignment of the protein sequences of PBIO3707 compared with the laboratory *E. coli* K-12 strain MG1655 (susceptible to colistin) revealed amino acid substitutions in PhoQ (Val386Leu) and EptA (Val336Met and Glu547Lys) that likely contributed to colistin resistance [37,38]. In addition, five strains (PBIO3678, PBIO3688, PBIO3691, PBIO3707, and PBIO3708) possessed heavy metal resistance genes, mainly associated with mercury resistance (*mer*; 4/5). The *K. pneumoniae* strain PBIO3691 demonstrated the largest set of heavy metal resistance genes, including resistance to arsenic (*ars*), copper (*pco*), mercury, and silver (*sil*).

### 2.3. Virulence Profiles of the Isolated Strains

**Figure 3:**
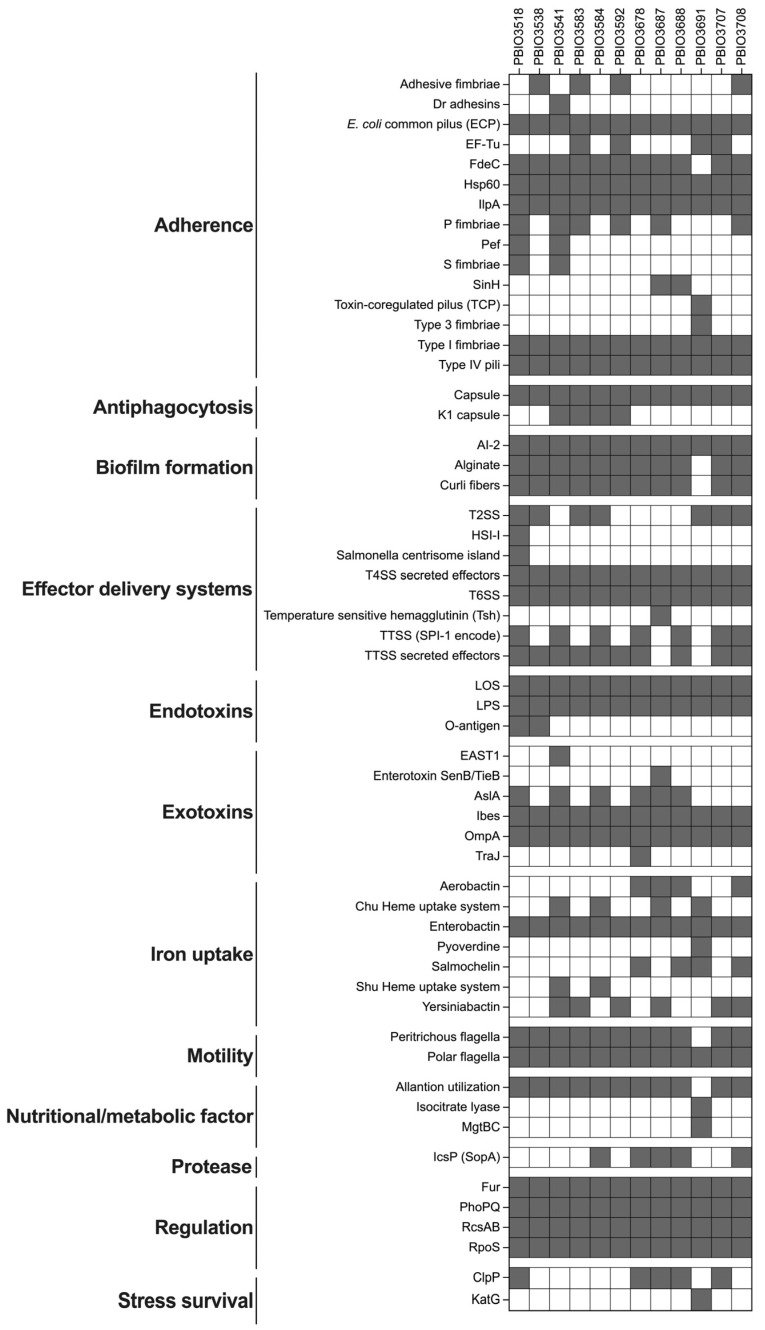
Overview of the virulence-associated genes (VAGs) carried by the 12 ESBL-producing strains. Predictions of VAGs (gray) were based on the alignment of sequences from the virulence factor database (threshold for coverage and identity, ≥65.0%).

As the combination of MDR and high levels of bacterial virulence seems to be a prominent feature of international high-risk clonal lineages [33,39,40], we examined the genes associated with *E. coli* and *K. pneumoniae* virulence using the virulence factor database [41] (Figure 3).

All 12 ESBL-producing strains carried different virulence-associated genes (VAGs), which were mainly associated with adherence, biofilm formation, iron uptake, bacterial motility, and toxin production, which suggests their pathogenic potential. For example, the P fimbriae adhesin cluster (*pap* operon; 50.0%, 6/12), the Dr family of adhesins (8.3%, 1/12), and Type I fimbriae (*fim*; 100.0%, 12/12) enable adhesion to uroepithelial cells and are thus often associated with strains that cause urinary tract infections [42].

Although PBIO3541 and PBIO3687 belong to the well-known ExPEC lineages ST38 and ST131, they carried enterotoxins typically found in obligately pathogenic *E. coli* that are uncommon in ExPEC strains. Here, PBIO3708 (ST58) showed the highest number of VAGs (*n* = 174 genes), followed by the two ST38 strains PBIO3541 (*n* = 165 genes) and PBIO3584 (*n* = 156 genes). In contrast, the *K. pneumoniae* strain PBIO3691 had a Kleborate virulence score of 0 because it lacked genes for yersiniabactin, colibactin, and aerobactin [43]. It carried a total of 102 VAGs, revealing the lowest level in our sample set.

### 2.4. Phylogenetic Relationship

**Figure 4:**
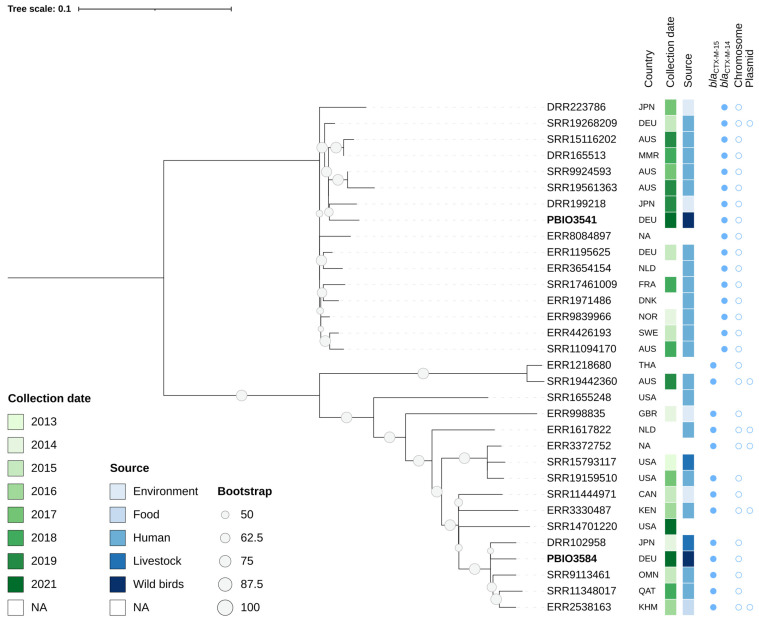
ST38 phylogeny. Phylogenetics revealed the close relationship of PBIO3541 and PBIO3584 with publicly available genomes (*n* = 30). The genomes included here were selected on the basis of the most shared k-mers using a Mash-based approach. The phylogenetic tree was constructed using a maximum likelihood-based approach and based on a core single nucleotide polymorphism alignment, with PBIO3541 as the reference. The tree was midpoint-rooted and the circumference of the circles on the branches indicates bootstrapping support of ≥50%. Labels indicate the accession numbers (except for PBIO3541 and PBIO3584, which are highlighted in bold). In addition, the three-letter country code indicates the sample’s origin (https://www.iso.org/obp/ui/#search, accessed on 19 September 2022). Different colors indicate the collection date and source. Further information includes the encoded ESBL genes and either their chromosomal or plasmid location. In the case of an unclear assignation, both the chromosome and plasmid locations are indicated (*n* = 6). NA, not available (i.e., no metadata provided); ST, sequence type.

Finally, to elucidate the phylogenetic relationships from a global perspective and to investigate the zoonotic potential of two of our most resistant and virulent strains, we constructed a phylogeny of 30 closely related, publicly available ST38 genomes in comparison with PBIO3541 and PBIO3584 (Figure 4).

The phylogeny showed two major clades with several subclades, including the two ST38 strains and publicly available genomes originating from humans, domestic and wild animals, food, and the environment. PBIO3541 was most closely related to a strain from a sewage treatment plant in Japan (DRR199218). In addition, we found related genomes from humans (SRR19561363, SRR9924593). Interestingly, SRR19561363 originated from a bloodstream infection of an Australian patient, pointing towards its clinical significance.

PBIO3584, as part of the second major clade, showed a close phylogenetic relationship to a strain derived from a fecal sample from a healthy cow (DRR102958 [44]). In addition to this apparent association with animal husbandry, PBIO3584 clustered with strains originating from hospitalized patients from different geographic locations (SRR9113461, SRR11348017 [45]) but not with PBIO3541, again indicating our strains’ zoonotic potential and the non-clonality of PBIO3584 and PBIO3541.

## 3. Discussion

Here, we isolated 12 ESBL-producing *E. coli* and *K. pneumoniae* strains from black-headed gull colonies breeding in two nature reserves in Western Pomerania in 2021. Their distribution is interesting, as 11 of the 12 strains originated from Böhmke Island and only one was from Riether Werder Island. Due to the shorter distance to the mainland and tourist hotspots (e.g., Neppermin), the birds from Böhmke Island apparently not only feed from the surrounding sea but also on remains from tourist sites, which might imply a higher risk of acquiring clinically relevant strains through closer contact with humans. In general, nature areas near human settlements are more affected by anthropogenic impacts, such as contaminated food/landfills, agriculture, and polluted surface waters that subsequently serve as the gulls’ habitat [46]. However, there is also evidence that individual birds move between the two locations [27], contributing to the occurrence of MDR bacteria in both colonies. Nevertheless, this is likely to be of minor importance, given the uneven distribution and unrelatedness of the strains across the islands.

ESBL-producing *Enterobacterales* have been detected worldwide in wildlife populations, including wild birds. For example, in Greece, 3.3% of fecal samples from wild birds contained ESBL-producing *E. coli* [47]. In pristine areas of Brazil, ESBL-producing *E. coli* were isolated from 2.4% of cloacal swabs from various seabird species, including the international high-risk clonal lineage ST131 [48], while a Nigerian study found a 100% rate in fecal samples from herons [49]. In the United Kingdom, 50% of herring gulls and black-backed gulls carried MDR pathogens [50], and a Swedish study found a prevalence of 8–55% in different Alaskan colonies, which was dependent on the predominant gull species at the sampling site [51]. However, the emergence of ESBL producers is not limited to wild bird populations. In Mecklenburg–Western Pomerania, we previously reported a rate of 1.2% in wild boars and wild ruminants [9], with a phylogenetic background similar to that of human isolates [11]. This underlines the potential of wildlife as a reservoir and a potential distributor of zoonotic pathogens.

The occurrence of five clinically relevant representatives belonging to international high-risk clonal lineages among only 12 ESBL-producing strains is remarkable. ST131 has not only been reported as the major *E. coli* clone in the clinical setting [32] but is often also found in veterinary, food, and environmental settings [52,53]. While previous studies have frequently found MDR ST58 strains in farm animals and, to a lesser extent, in wild birds [54,55] and as contaminants of retail meat [21], ST38 has been detected in several animal species, including broilers, rats, and black-headed gulls [12,56], with the latter matching our results. ST744 and ST617 have also been previously detected in German birds of prey and in rooks wintering in Europe, fitting into the pattern of the locally settled wild birds [16,57]. Taken together, our results and those of others highlight the omnipresence of international high-risk clonal *E. coli* lineages in wildlife species, particularly in wild birds. Moreover, ST290 represents a phylogenetic background of MDR *K. pneumoniae* that is not only present in wildlife [58] but has previously led to lethal outbreaks in human settings [59,60]. Other *K. pneumoniae* STs have been detected in wild birds, such as ST37 in Canadian ravens and ST548 in Australian silver gulls [61,62]. Thus, the occurrence of ST290 seems to be new in wildlife; however, other resistant *K. pneumoniae* STs are a known phenomenon.

A major contributor to the global dissemination of bacterial pathogens is sewage water, which originates from private and public facilities, including hospitals, and which is insufficiently treated before entering surface waters such as rivers and seas [63]. In addition, contaminated manure on fertilized agricultural fields exacerbates this situation [64,65]. Spillover to environmental habitats, where the wildlife acquires MDR pathogens and migrating wild birds in particular carry them for long distances, happens frequently [66]. This leads to the global dissemination of MDR bacteria, even in the most remote regions, again supporting the necessity of the One Health approach. None of the major public, veterinary, and environmental health disciplines should be considered separately [67].

Another interesting finding in our study was the chromosomal location of the ESBL genes in the four strains. It is well known that mostly plasmids drive the emergence of cephalosporine resistance in *Enterobacterales* [31]. Note that both ST38 strains carried the *bla*_CTX-M_ genes chromosomally, which is consistent with our previous results, where the ST38 *E. coli* strains also originated from wild birds [19]. Chromosomal integration of previous accessory features represents a stable “fixation” of resistance for the bacterium, as the gene can no longer be lost as easily through plasmid loss [68], potentially leading to long-term benefit. Apart from non-susceptibility to antibiotics, bacterial heavy metal resistance occurs increasingly frequently, mostly driven by anthropogenic pollution and enhanced use, for example, in livestock feed [69]. In the case of the co-localization of heavy metal and antibiotic resistance genes on mobile genetic elements, heavy metals may promote the selection of MDR *E. coli* and *K. pneumoniae* in the environment [70], potentially leading to their even more accelerated dissemination.

Finally, our genomic analysis revealed that the ESBL-producing strains carried multiple virulence factors, likely providing an advantage in addition to antimicrobial resistance. For example, the ability to adhere to surfaces and form biofilms is important to help bacteria resist external stressors such as antibiotic treatment, UV radiation, and low temperatures [71]. Note, however, that we did not perform phenotypic virulence assays, which leads to a limited understanding of the bacteria’s pathogenicity in vivo. We will address these experiments in the future. Nevertheless, it is well known that successful international high-risk clonal lineages such as the identified ST38, ST58, and ST131 usually combine MDR with high-level phenotypic virulence [22]. This, plus the fact that we detected germane strains that were clinically relevant, suggests their zoonotic and pathogenic potential, at least for ST38 and likely also for the other clonal lineages.

In summary, here, the occurrence of multiple high-risk clonal lineages in wild birds demonstrates the importance of One Health as a paradigm approach that recognizes the interdependence of human, animal, and environmental health. Both humans and animals depend on sustainable ecosystems, where small changes impact the health of all, which is also true in a global context.

## 4. Materials and Methods

### 4.1. Sampling Strategy and Bacterial Isolation

During the 2021 breeding season (May–June), we collected 339 different samples from two black-headed gull colonies on Riether Werder and Böhmke Islands, both located in Western Pomerania, Germany (Figure 1). Individual cloacal swabs (Sarstedt, Nümbrecht, Germany) were taken from adults and newly hatched nestlings. At Böhmke Island, 100 cloacal swabs were taken from adult birds and 99 swabs from nestlings, while at Riether Werder, 111 swabs were obtained from adults and none from nestlings. In addition, collective fecal samples were collected by pooling 5–10 individual fecal samples in 50 mL polypropylene tubes (Sarstedt, Nümbrecht, Germany). We thus included 14 collective fecal samples from Böhmke Island and 15 from Riether Werder Island. All samples were screened for ESBL-producing *E. coli* and *K. pneumoniae*. For this purpose, some material from the cloacal swabs or the mixed collective fecal sample was added individually to 5 mL of lysogeny broth (LB; Carl Roth, Karlsruhe, Germany) supplemented with 4 μg/mL cefotaxime (Cayman Chemical, Ann Arbor, MI, USA) and incubated overnight under shaking conditions (130 rpm) at 37 °C. One hundred microliters of the cultures was then plated on chromogenic agar plates (CHROMagar orientation, Mast Diagnostica, Reinfeld, Germany), spiked with cefotaxime. The plates were incubated overnight at 37 °C. According to the manufacturer’s protocol, the *E. coli* colonies were pinkish-purple, while the *K. pneumoniae* colonies had a metallic blue color. Subsequently, species identity was confirmed with API ID strips (bioMérieux, Marcy-l’Étoile, France). All confirmed strains were stored at −80 °C in LB containing 20% (*v*/*v*) glycerol (anhydrous; Merck, Darmstadt, Germany). Fresh overnight cultures were streaked on LB agar plates before use.

### 4.2. Phenotypic Antimicrobial Susceptibility Testing

We performed phenotypic antimicrobial susceptibility testing to investigate the occurrence of additional clinically relevant resistance phenotypes. Briefly, a single colony was picked and streaked on chromogenic agar plates containing antibiotics at their clinical breakpoint concentrations according to the published breakpoints of the Clinical and Laboratory Standards Institute [72]. We used the antibiotics tetracycline (16 μg/mL; VWR International, Radnor, PA, USA), gentamicin (2 μg/mL; Carl Roth, Karlsruhe, Germany), and ciprofloxacin (1 μg/mL; VWR International, Radnor, PA, USA). The minimum inhibitory concentration of colistin was determined using the MICRONAUT MIC-Strip Colistin Assay (Merlin Diagnostika, Bornheim, Germany) according to the manufacturer’s instructions.

### 4.3. Whole-Genome Sequencing

Total DNA was extracted using the MasterPure DNA Purification Kit for Blood, Version 2 (Lucigen, Middelton, WI, USA), according to the manufacturer’s instructions. The isolated DNA was quantified fluorometrically using the Qubit 4 fluorometer and the corresponding dsDNA HS Assay Kit (Thermo Fisher Scientific, Waltham, MA, USA). DNA was shipped to the SeqCenter (Pittsburgh, PA, USA) and, following library preparation using the Illumina DNA Prep Kit and IDT 10 bp UDI indices (Illumina, San Diego, CA, USA), whole-genome sequencing was carried out on an Illumina NextSeq 2000, producing 2 × 151 bp reads. Demultiplexing, quality control, and adapter trimming were performed using bcl-convert Version 3.9.3 [73].

### 4.4. Sequence Assembly and Genomic Analyses

Short-read data were processed using BBDuk from BBTools Version 38.95 (https://sourceforge.net/projects/bbmap/, accessed on 8 March 2022) to trim the adapters, filter possible PhiX contaminants, and conduct further quality and polymer trimming. Quality control (QC) of the provided (raw) and processed (trimmed) reads was performed using FastQC Version 0.11.9 (https://www.bioinformatics.babraham.ac.uk/projects/fastqc/, accessed on 8 March 2022). Trimmed reads were assembled using shovill Version 1.1.0 (https://github.com/tseemann/shovill, accessed on 8 March 2022) with SPAdes Version 3.15.3 [74]. An additional polishing step (besides the one implemented in the shovill assembly pipeline) was performed by first mapping the trimmed reads to the assembly using BWA Version 0.7.17 [75]. The alignment files were then converted to binary format and sorted, and the duplicate reads were marked with SAMtools Version 1.14 [76]. Finally, the draft contigs were corrected using Pilon Version 1.24 [77]. Genome completeness and contamination were assessed using CheckM Version 1.1.3 [78]. Prokka Version 1.14.6 [79] was used to automatically annotate the draft assembly. Genomic analyses including in silico multilocus sequence typing, serotype prediction, and detection of antibiotic resistance and virulence features were performed using mlst Version 2.19.0 (https://github.com/tseemann/mlst, accessed on 11 March 2022), with the PubMLST [80] database and Enterobase [81]; ABRicate Version 1.0.0 (https://github.com/tseemann/abricate, accessed on 11 March 2022), with the PlasmidFinder [82] and VFDB databases [41]; and AMRFinderPlus Version 3.10.20 with the database version 2021-12-21.1 [30]. Assignment to the phylogroups was performed using ClermonTyping Version 20.03 [83]. In addition, PBIO3691 was analyzed using Kleborate Version 2.2.0 [43] with Kaptive Version 2.0.0 [84].

To determine whether the *bla*_CTX-M_ genes were encoded chromosomally or in plasmids, contigs were compared with the NCBI Nucleotide Database using blastn Version 2.13.0. The top 50 hits (to different subjects, where available) were used to manually determine the origin of the gene. Queries with all subjects annotated as chromosomes were classified as having a chromosomal origin. Accordingly, queries with hits to subjects annotated as plasmids and to subjects annotated as chromosomes were classified as ambiguous.

### 4.5. Phylogeny

For the creation of a core single nucleotide polymorphism (SNP)-based phylogeny of closely related isolates, Enterobase [81] was searched for *E. coli* ST38 strains (*n* = 2910) and the available assemblies were downloaded on 5 July 2022. Mash Version 2.3 [85] was used to generate sketches (sketch size, 1,000,000) of all downloaded genomes and the draft genomes of PBIO3541 and PBIO3584. Afterwards, the pairwise distance between PBIO3541 or PBIO3584 and the sketch archive was estimated. For a better overview, the 15 assemblies with the shortest distances, excluding the hits of PBIO3541 or PBIO3584 to itself (most shared k-mers), for which paired Illumina read data were available were selected. The data were downloaded from the public European Nucleotide Archive SRA FTP server (ftp://ftp.sra.ebi.ac.uk/vol1/, accessed on 25 July 2022). For these accessions, read trimming, genome assembly, and analyses were performed as described above. The trimmed reads were mapped against the draft genome of PBIO3541 using snippy Version 4.6.0 (https://github.com/tseemann/snippy, accessed on 27 July 2022) to create a whole-genome alignment of 32 sequences. The alignment was processed using Gubbins Version 3.2.1 [86] to filter out regions with SNPs that were likely the result of recombination. The alignment of sequences was processed using SNP-sites Version 2.5.1 [87] to retain only the alignment positions containing A, C, G, or T exclusively. A maximum likelihood tree was inferred with RAxML-NG Version 1.1.0 [88] using GTR+G by first parsing the alignment. The final alignment (containing 32 sequences and 1261 sites) was then processed by searching 500 parsimonious trees and 500 random starting trees, and performing 1000 bootstrap repeats. The best-scoring maximum likelihood tree was midpoint-rooted by iTOL Version 6.5.7 [89] and visualized with the bootstrap support values and metadata.

## Figures and Tables

**Table 1:** Overview of the 12 ESBL-producing strains studied for their phylogenetic background, their origin, the replicon types, and the encoded ESBL gene.

Strain	Species	ST ^a^	Phylogroup	Island ^c^	Source	Replicon Type	ESBL Gene
PBIO3518	*E. coli*	34	A	RW	Collective feces	FII, I1, X1/4, Y	*bla*_CTX-M-14_
PBIO3538	*E. coli*	**58**	B1	B	Collective feces	Q2	*bla*_CTX-M-15_
PBIO3541	*E. coli*	**38**	D	B	Collective feces		*bla*_CTX-M-14_
PBIO3583	*E. coli*	453	B1	B	ICS ^d^ nestling	X4/8, Y	*bla*_CTX-M-15_
PBIO3584	*E. coli*	**38**	D	B	ICS nestling		*bla*_CTX-M-15_
PBIO3592	*E. coli*	453	B1	B	ICS nestling	Y	*bla*_CTX-M-15_
PBIO3678	*E. coli*	**744**	A	B	ICS nestling	FIA/B/C, FII, Q1, X1	*bla*_CTX-M-55_
PBIO3687	*E. coli*	**131**	B2	B	Collective feces	FIA/B, FII	*bla*_CTX-M-27_
PBIO3688	*E. coli*	**617**	A	B	Collective feces	FIB, FII, I1, Q1	*bla*_CTX-M-15_
PBIO3691	*K. pneumoniae*	**290**	NA ^b^	B	Collective feces	FIB, FII	*bla*_CTX-M-15_
PBIO3707	*E. coli*	1598	A	B	ICS adult	FII, R	*bla*_CTX-M-15_
PBIO3708	*E. coli*	**58**	B1	B	ICS adult	FIB, FII, I1, Q1	*bla*_CTX-M-32_

^a^ ST, sequence type. Pandemic clonal lineages are highlighted in bold. ^b^ NA, not applicable. ^c^ B, Böhmke Island; RW, Riether Werder Island. ^d^ ICS, individual cloacal swab.

## Data Availability

The data for this study have been deposited in the European Nucleotide Archive (ENA) at EMBL-EBI under the accession number PRJEB55873 (https://www.ebi.ac.uk/ena/browser/view/PRJEB55873, accessed on 9 September 2022).
